# Titin: a phenotype-genotype descriptive comparison of dilated cardiomyopathy

**DOI:** 10.1186/1532-429X-16-S1-O89

**Published:** 2014-01-16

**Authors:** Arun J Baksi, Angharad M Roberts, James S Ware, Ankur Gulati, Rachel J Buchan, Roddy Walsh, Shibu John, Samuel Wilkinson, Aamir Ali, Ravi G Assomull, Paul J Barton, Sanjay K Prasad, Dudley J Pennell, Stuart A Cook

**Affiliations:** 1 NIHR Cardiovascular Biomedical Research Unit at Royal Brompton & Harefield NHS Foundation Trust, London, UK; 2 National Heart and Lung Institute, Imperial College London, London, UK

## Background

Cardiovascular magnetic resonance (CMR) imaging is the gold standard imaging modality for characterization of dilated cardiomyopathy (DCM). We recently identified genetic variants that truncate the giant protein Titin (TTN) accounting for up to 25% of DCM cases: a discovery that will increase the utility of genetic testing and family screening in DCM. However, the clinical consequences of TTN truncating variants (TTNtv) are unknown. Here, we integrated the power of CMR and capacity of next generation sequencing (NGS) to assess the relationship between genotype and phenotype in a non-ischemic DCM population.

## Methods

319 patients referred for characterization of DCM were recruited and underwent quantitative CMR study with detailed phenotyping (Siemens Avanto or Siemens Sonata 1.5T scanners). Additional clinical data were collected. TTN genotypes were determined by target enrichment using SureSelect and NGS sequencing using the SOLiD 5500 platform.

## Results

TTN truncating variants (TTNtv) were identified in 13% (n = 42) of cases, the commonest genetic cause of DCM. Table 1 compares the CMR and clinical characteristics of TTNtv-positive and TTNtv-negative cohorts. TTNtv-positive DCM patients had lower left ventricular (LV) ejection fraction (EF) (p = 0.02), thinner LV walls (p < 0.02) and higher incidence of sustained ventricular tachycardia (VT) (p = 0.001) which was independent of EF. No statistically significant difference in the prevalence of midwall fibrosis existed between the two groups. Multivariate linear regression models that considered age, sex, TTNtv presence and position (distance from the protein N-terminus) indicated that TTNtv position was significantly correlated with biventricular ejection fraction and indexed stroke volumes such that more distal truncations had worse indices of cardiac function. For example, a C-terminal TTNtv would be associated with an absolute reduction in LV EF of 18 ± 7%, (p = 0.005), as compared with an N-terminal TTNtv.

**Table 1:** CMR and Clinical Characteristics of DCM patients with and without TTNtv

	TTN truncation negative (n = 277)	TTN truncation positive (n = 42)	p-value
**CMR data**			
LV EDVi (mL/m^2^)	136 ± 38	140 ± 34.5	0.34
LV ESVi (mL/m^2^)	87.5 ± 39.1	96.5 ± 36.7	0.07
LV SVi (mL/m^2^)	48.2 ± 12.9	43.4 ± 13.6	0.03*
LV EF (%)	37.5 ± 12.2	32.7 ± 13	0.02*
RV EDVi (mL/m^2^)	89.4 ± 24.7	90.7 ± 25.5	0.71
RV ESVi (mL/m^2^)	45.2 ± 22.3	50.6 ± 24.9	0.15
RV SVi (mL/m^2^)	44.4 ± 12.6	40.1 ± 15.6	0.09
RV EF (%)	51.6 ± 14.1	45.5 ± 15.5	0.04*
LAVi (mL/m^2^)	63.8 ± 26.8	66.7 ± 25.7	0.30
LVMi (g/m^2^)	95.4 ± 27.6	85.5 ± 18.6	0.03*
Lateral WTi (mm/m^2^)	3.1 ± 0.7	2.8 ± 0.7	0.002**
Septal WTi (mm/m^2^)	4.2 ± 0.9	3.9 ± 0.8	0.02*
Maximum WTi (mm/m^2^)	5.3 ± 1.0	4.9 ± 0.9	0.01*
Mid-wall fibrosis present	94/270	13/42	0.73
**Clinical data**			
Age at Diagnosis (years)	53.4 ± 13.3	50.4 ± 13.9	0.29
NYHA status 1/2/3/4	116/103/36/1	19/16/4/1	0.43
Sustained VT	20/97	9/14	0.001**
Conduction Disease	82/227	9/37	0.19
Family history of DCM	24/218	10/38	0.018*

Values are means ± SD for continuous data. TTN, Titin; LV, left ventricular; RV, right ventricular; EDVi/ESVi, end diastolic/systolic volume indexed to body surface area (BSA); SVi, stoke volume indexed to BSA; EF, ejection fraction; LAVi, left atrial volume indexed to BSA; LVMi, LV mass indexed to BSA; WTi, wall thickness indexed to BSA; VT, ventricular tachycardia; NYHA, New York Heart association functional class. *p < 0.05, **p < 0.01

## Conclusions

These data highlight the prevalence of TTNtv in this unselected DCM cohort and suggest that TTNtv-positive DCM patients may benefit from tailored clinical management. Sustained VT, LV wall thickness and LVEF influence DCM outcomes. The increased risk of sustained VT in TTNtv-positive DCM may warrant a lower threshold for device therapy, as recommended for DCM caused by LMNA mutations. As distal TTNtv were associated with worse LV function, more aggressive clinical management may be appropriate in patients carrying these variants. The phenotypic expression of genetic substrates can be highly variable, and CMR is central to the accurate phenotypic description of this common yet heterogenous condition. Enhanced molecular understanding may lead to strategies to alter disease course beyond current pharmacological and device based therapy.

## Funding

This research was supported by the NIHR Cardiovascular Biomedical Research Unit at Royal Brompton & Harefield NHS Foundation Trust and Imperial College London, the British Heart Foundation (BHF) UK, the Medical Research Council (MRC) UK, the Wellcome Trust UK, Leducq Foundation, Heart Research UK, CORDA, the NMRC Singapore and Rosetrees Trust.

